# Identifying haemochromatosis patients with C282Y homozygosity from inpatient electronic patient records in England using a novel algorithm: a retrospective observational study

**DOI:** 10.1136/bmjopen-2024-089369

**Published:** 2025-02-18

**Authors:** Prabhsimran Singh, Charles Millson, Chao Huang, Robert J Driver

**Affiliations:** 1 University of York, York, UK; 2 Department of Hepatology, York and Scarborough Teaching Hospitals NHS Foundation Trust, York, UK; 3 Hull York Medical School, York, UK; 4 Institute of Clinical and Applied Health Research, University of Hull, Hull, UK

**Keywords:** Hepatobiliary disease, Hepatology, Other metabolic, eg iron, porphyria, Electronic Health Records, International health services

## Abstract

**Abstract:**

**Introduction:**

Hereditary haemochromatosis (HH) is the most common genetic condition among populations of northern European ancestry, but it does not have a specific International Classification of Diseases 10th revision (ICD-10) diagnosis code. HH is commonly assigned the ICD-10 code E83.1 defined as ‘disorders of iron metabolism’. However, the E83.1 diagnosis code is also applied to patients with transfusion-related iron overload and hyperferritinaemia from non-iron loading conditions. Venesection is the main treatment option for patients with HH and is assigned the Office of Population, Census and Surveys Classification of Interventions and Procedures, 4th revision (OPCS-4) code X36.2. We aimed to develop a novel algorithm to identify haemochromatosis patients with C282Y homozygosity from electronic patient records (EPR) using ICD-10 and OPCS-4 codes.

**Methods:**

The Hospital Episode Statistics Admitted Patient Care (APC) database was used to identify all patients with the ICD-10 code E83.1 from 1 April 2018 to 31 March 2023 in our NHS Trust. Case notes were reviewed to evaluate the presence of HH, genotype, medical history and associated procedures. Algorithms were generated using Stata MP V.18 by applying a combination of ICD-10 and OPCS-4 codes to all patients with the diagnosis code E83.1 including those without HH gene mutations.

**Results:**

A total of 9264 patient episodes were identified from the HES APC database corresponding to 787 unique patients: 479 (60.9%) C282Y homozygous, 107 (13.6%) C282Y/H63D compound heterozygotes, 42 (5.3%) other HH genetic mutations and 159 (20.2%) without any HH gene mutations. Six algorithms were developed to identify patients with HH within inpatient EPR, all of which showed improved positive predictive value (PPV) compared with the baseline cohort. The application of the OPCS-4 code for venesection (X36.2) in five of the algorithms resulted in large improvements in specificity and increased all their PPVs to >70%. Algorithms 4 and 6 had the best PPV at 74.3% and 74.4%, respectively, and included the removal of patients with ICD-10 codes associated with transfusion-related iron overload and other conditions treated with venesection.

**Conclusion:**

These novel algorithms represent a more reliable method to detect haemochromatosis patients with C282Y homozygosity from EPR than the single ICD-10 code E83.1. It may be applied to large administrative datasets for investigation of HH in population studies.

**STRENGTHS AND LIMITATIONS OF THIS STUDY**

- Case note review undertaken for all patients assigned the International Classification of Diseases 10th revision (ICD-10) diagnosis code E83.1 ‘disorders of iron metabolism’ to determine their cause of iron overload.
- Methodical generation of algorithms considering various other conditions which influence the ICD-10 code E83.1.
- This study has not been externally validated in England or other healthcare settings.
- Our study relied on inpatient coding of venesections only to capture the performance of algorithms; however, venesection is undertaken in outpatient clinics in some National Health Service Trusts.
- Some patients undergo maintenance treatment by donating blood or are treated with oral iron chelation only and thus may not be captured by the Hospital Episode Statistics Admitted Patient Care database.

## Introduction

 Hereditary haemochromatosis (HH) is an inherited disorder of iron homeostasis characterised by excess iron deposition in various organs such as the liver, pancreas, heart and pituitary gland. HH is the most common genetic condition among populations of northern European ancestry.^1^

Mutation to the *HFE* gene on chromosome 6 is the most common cause of HH, giving rise to the presence of the C282Y variant and H63D variant. Those with two copies of the C282Y mutation (C282Y homozygotes) are at the highest risk of iron overload and developing clinical manifestations of the disease.^2^ 1 in 156 individuals are estimated to be homozygous for the C282Y mutation in the UK; however, not all of them will develop HH due to the variable biochemical and clinical penetrance seen with this condition.^3^ Individuals who have one copy of the C282Y and one copy of the H63D gene are termed compound heterozygotes. These individuals are less likely to have excess iron deposition in their organs and morbidity or mortality related to HH unless in the presence of cofactors such as alcohol.^3^,^5^ Mutations to other genes (non-*HFE*) that cause iron overload such as haemojuvelin, hepcidin antimicrobial peptide, transferrin receptor 2 and *SLC40A1* (ferroportin disease) are all rare.^6^

Diagnostic and procedure classification systems are used as part of routine clinical care to provide a uniform record of healthcare data as well as mortality data. The International Classification of Diseases (ICD) developed by the WHO and currently in its 11th iteration (ICD-11), is the most commonly used disease classification system. The Office of Population, Census and Surveys Classification of Interventions and Procedures, currently in its fourth version (OPCS-4), is the classification system used in the UK for recording medical procedures. In the USA, coding is crucial for the billing process.^7^ Increasingly, large data sets using ICD and OPCS coding are used for clinical research to understand prevalence, outcomes, regional variation and mortality for various medical conditions.^8^

Despite the prevalence of HH, this condition does not have a specific ICD-10 diagnosis code making identification of HH patients challenging within electronic patient records (EPR). Surprisingly, the ICD-11, which came into effect recently after several years of developments and revisions, does not have a specific code for HH either.^9^ Thus, when using ICD-10, HH is often coded as E83.1 for ‘disorders of iron metabolism’. Venesection is the main treatment option for patients with HH and is assigned the OPCS-4 code X36.2.

The imprecision of the E83.1 code allows a variety of non-HH conditions such as secondary iron overload and hyperferritinaemia to be incorporated. Specifically, patients who require frequent blood transfusions can develop secondary iron overload from haematological conditions such as thalassaemia, myelodysplasia, aplastic anaemia and acute myeloid leukaemia.^10^ In addition, an elevated serum ferritin which, in HH patients denotes the degree of iron overload, will also be raised in any state of inflammation because it is an acute phase protein. Thus, patients who do not have HH but do have a raised ferritin, perhaps due to acute infection, chronic inflammation, excess alcohol consumption, steatotic liver disease or liver cirrhosis regardless of aetiology,^11^ may also attract the ICD-10 code E83.1. Indeed, a small study in a UK district general hospital confirmed the E83.1 code was assigned to 31% of patients with hyperferritinaemia.^12^

Although the ICD-10 coding has been developed by the WHO, some nations have modified this to suit their healthcare systems, policy, resource management and billing processes with some variation between the categories and subcategories.^13^ In the USA, the ICD-10 clinical modification (ICD-10-CM), used since 2015, is an adaptation of the ICD-10 with more refinements to assist with the billing process. Given the greater precision within the ICD-10-CM, the code E83.110 is specific for HH. Those with iron overload secondary to chronic blood transfusions are coded as E83.111. A precise code for HH was also used in ICD-9 coding in the USA which allowed accurate identification of HH patients to understand the healthcare utilisation and economic burden to this group of patients.^14^ These more specific codes are not currently used in the UK.

The Hospital Episode Statistics (HES) admitted patient care (APC) database records all inpatient admissions to any hospital in England which is uploaded onto a national database. Designated clinical coders interpret medical diagnoses and procedures following each admission and translate this into organised ICD-10 and OPCS-4 codes which are inputted into the HES database. A previous study in England investigating admission trends related to HH using the HES database used the ICD-10 code E83.1 to identify the HH population. The authors acknowledged the assumption in their study that the E83.1 diagnosis code equated to a diagnosis of HH but inferred this was unlikely to affect their conclusions as the inclusion of admissions for non-HH conditions would be small.^15^ The UK General Practice Research Database has been previously interrogated to study the prevalence of HH, but this database uses Read Codes, which are a historical coded thesaurus of clinical terminology specific for HH (Read Code C350000).^16^ Despite the lack of a specific ICD-10 code, various studies using large national databases have relied on E83.1 to characterise HH patients without accounting for its limitations.^17^,^19^

The aim of this study is to develop a nuanced algorithm using ICD-10 and OPCS-4 codes to identify C282Y homozygotes with an established diagnosis of haemochromatosis from EPR. Accurately identifying this group of patients in large data sets is crucial to understand their healthcare utilisation, the prevalence of organ dysfunction and risk of hepatocellular carcinoma to guide healthcare practitioners and organisations in developing guidelines, public health strategies for screening and areas to focus further research.

## Methods

### Study population

All patients aged ≥18 years with an ICD-10 code E83.1 (‘disorders of iron metabolism’) contained in the HES APC database for episodes from 1 April 2018 to 31 March 2023 in York and Scarborough Teaching Hospitals NHS Foundation Trust (Y&S) were included in our study (baseline cohort). The study period was limited to 5 years reflecting the Trust’s policy on data retention. ICD-10 diagnosis code E83.1 was used to define the study population as the closest ICD-10 code assigned to patients with HH. When patients undergo venesection as a day case, an inpatient episode is generated and ICD-10 and OPCS-4 codes are recorded in the HES APC database. Additional inpatient episodes unrelated to venesection would also be captured in this database, so HH patients who had not undergone venesection during the study period could also be identified. However, HES outpatient data sets could not be incorporated owing to their poverty in coding and lack of validity.^20^

### Identification of hereditary haemochromatosis within the baseline cohort

A case note review was undertaken by PS to determine the diagnosis of haemochromatosis. Only haemochromatosis patients with C282Y homozygosity were defined as having haemochromatosis for our study. Several current international guidelines, including the European Association for the Study of the Liver and American College of Gastroenterology acknowledge that patients with compound heterozygosity (C282Y/H63D) may have raised iron indices but state that they are at a lower risk of morbidity and mortality related to iron overload unless in the presence of cofactors.^1 6^ The UK BioBank database reviewed 10 701 compound heterozygotes and did not find increased mortality in this group compared with those without any *HFE* mutations.^3^ Non-*HFE* mutations are rare and were considered unlikely to significantly influence the results of our study.

For each patient in the baseline cohort, clinical letters, inpatient discharge summaries, emergency department visits, biochemical parameters and genetic test results were examined to determine the presence of C282Y homozygosity and confirm a diagnosis of haemochromatosis. Some patients labelled as HH on clinic letters did not have a genetic test result available due to either historical testing or having previously moved from a different NHS Trust. These patients were labelled as ‘HH gene unknown’ (table 1).

**Table 1:** Diagnosis of patients with ICD-10 diagnosis code E83.1 in the Y&S cohort

Diagnosis/genotype	TotalN (%)	X36.2 presentN (%)	X36.2 absentN (%)
HH-related genes			
C282Y/C282Y	479 (60.90)	416 (52.85)	63 (8.05)
C282Y/H63D	107 (13.60)	81 (10.30)	26 (3.30)
C282Y/x*	17 (2.16)	7 (0.89)	10 (1.27)
H63D/H63D	5 (0.64)	5 (0.51)	0 (0)
H63D/x*	5 (0.64)	4 (0.51)	1 (0.13)
HH gene unknown	10 (1.3)	10 (1.3)	0 (0)
Rarer mutations†	5 (0.64)	5 (0.64)	0 (0)
Other diagnoses			
Transfusion iron overload	17 (2.16)	6 (0.76)	11 (1.40)
Hyperferritinaemia	73 (9.28)	24 (3.05)	49 (6.23)
Polycythaemia vera	10 (1.27)	10 (1.3)	0 (0)
Secondary polycythaemia	4 (0.51)	4 (0.51)	0 (0)
Porphyria cutanea tarda	4 (0.51)	3 (0.38)	1 (0.13)
Incorrect coding	51 (6.48)	6 (0.76)	45 (5.72)

* x denotes heterozygosity.

† Includes patients with the following mutations: E277K, S65C, haemojuvelin (), hepcidin antimicrobial peptide (), transferrin receptor 2 (TFR2), SLC40A1 (ferroportin disease) and bone morphogenetic protein 6 (BMP6).

HHhereditary haemochromatosisICDInternational Classification of Diseases

Case notes were further interrogated in those without iron overload-related genetic mutations or a diagnosis of HH but undergoing regular venesections to confirm the diagnosis of polycythaemia vera, secondary polycythaemia or porphyria cutanea tarda (Group 1 in table 2). Patients without a positive genetic mutation related to iron overload or a ‘Group 1 diagnoses’ but who have a haematological condition were categorised ‘as transfusion-related iron overload’ (Group 2 in table 2). Among the remaining patients, the diagnosis of ‘hyperferritinaemia’ was applied to those who did not meet any of the above criteria but did have a condition associated with high serum ferritin and laboratory confirmed elevated serum ferritin level. ‘Incorrect coding’ was designated to the remaining patients.

**Table 2:** ICD-10 diagnosis codes for non-HH conditions requiring venesection and haematological conditions associated with transfusion-related iron overload

Category	ICD-10 code	Description
Group 1 (G1)(Non-HH conditions requiring venesection)	D45	Polycythaemia vera
	D75.1	Secondary polycythaemia
	E801	Porphyria cutanea tarda
Group 2 (G2)(Haematological conditions associated with transfusion-related iron overload)	D46	Myelodysplastic syndromes
	D47.4	Osteomyelofibrosis
	D55	Anaemia due to enzyme disorders
	D56	Thalassaemia
	D57	Sickle cell disorders
	D58	Other hereditary haemolytic anaemias
	D59	Acquired haemolytic anaemia
	D60	Acquired pure red cell aplasia
	D61	Other aplastic anaemias
	C91	Lymphoid leukaemia
	C92	Myeloid leukaemia
	C93	Monocytic leukaemia
	C94	Other leukaemias of specified cell type
	C95	Leukaemia of unspecified cell type

HHhereditary haemochromatosisICDInternational Classification of Diseases

### Algorithm development to identify haemochromatosis patients with C282Y homozygosity

Following the case note review, the prevalence of haemochromatosis patients with C282Y homozygosity in the baseline cohort defined by ICD-10 code E83.1 was only 60.9% (479/787). A series of algorithms were developed to test their performance against the baseline cohort (algorithm 0). ICD-10 codes for non-HH conditions requiring frequent venesection (G1 in table 2) were included in several algorithms to improve their performance by eliminating patients with these conditions. In addition, ICD-10 codes of haematological diagnoses (G2 in table 2) that require regular blood transfusions were also used. Algorithm 1 removes those patients with diagnoses in G1 and G2 from algorithm 0 (baseline cohort).

Venesection (OPCS-4 code X36.2) was applied in the algorithm development to identify HH patients undergoing treatment. Also, the addition of X36.2 aimed to eliminate those with hyperferritinaemia or incorrect coding as these patients are unlikely to undergo this treatment. Venesection frequencies of ≥5 and ≥10 episodes were also included in the algorithm generation to reflect HH treatment which requires weekly to biweekly venesection during the induction phase and between two and six times a year during the maintenance phase.^21^ At least 10 venesection episodes were chosen as one of the algorithms to reflect the minimum number of venesections that would have been undertaken by a patient undergoing maintenance therapy during the 5-year study period. An algorithm with five or more venesections was also generated to account for patients who during the study period may have become frailer and hence stopped treatment, patients who have paused venesections to prioritise treatment of other medical conditions such as cancer or patients who were non-adherent and only attending for venesections sparingly. Table 3 demonstrates the algorithms that were developed using a combination of ICD-10 and OPCS-4 codes as justified above.

**Table 3:** Algorithms developed using ICD-10 and OPCS-4 coding

Algorithm	ICD-10 and OPCS-4 coding	Description
0	E83.1	Baseline cohort of E83.1 only from HES APC database
1	E83.1 – (G1) – (G2)	Patients with G1 and G2 ICD-10 codes removed from algorithm 0
2	E83.1 + X36.2	Only patients with venesection procedure code X36.2 from algorithm 0 included
3	E83.1 + X36.2 – (G1)	Includes patients with venesection code and removes those with G1 ICD-10 codes
4	E83.1 + X36.2 – (G1) – (G2)	Similar to algorithm 3 but also removes patients with G2 ICD-10 codes
5	E83.1 + (≥5 × 36.2 episodes) – (G1) – (G2)	Only includes those with five or more episodes of venesection OPCS-4 code and eliminates patients with G1 and G2 ICD-10 codes
6	E83.1 + (≥10 × 36.2 episodes) – (G1) – (G2)	Only includes those with 10 or more episodes of venesection OPCS-4 code and eliminates patients with G1 and G2 ICD-10 codes

APCadmitted patient careHESHospital Episode StatisticsICDInternational Classification of DiseasesOPCSOffice of Population, Census and Surveys Classification of Interventions and Procedures

### Data analysis

Stata MP V.18 was used for the data management and analysis. Demographic characteristics and the diagnosis or genotypes for all patients were reported via descriptive statistics. Association between age group (<60 or ≥60) and presence of C282Y homozygosity was evaluated by χ^2^ test. Based on the relevant ICD-10 and OPCS-4 codes, six algorithms were developed as described in table 3 and applied against the baseline cohort. The sensitivity, specificity, positive predictive value (PPV) and negative predictive value were calculated and reported with 95% CIs for performance comparison among the developed algorithms.

### Patient and public involvement

Patients and public were not involved in this study.

## Results

A total of 9264 patient episodes were included corresponding to 787 patients with a median age of 63 (IQR 53–74). 58.4% (460/787) of patients were men. Demographic characteristics are included in online supplemental table 1. Those under the age of 60 were more likely to have C282Y homozygosity compared with those aged greater than 60 (p<0.01, χ^2^ test).

Evaluation of case notes revealed only 479 (60.9%) of the 787 patients with ‘disorders of iron metabolism’ (E83.1 code) were C282Y homozygous with 107 (13.6%) compound heterozygotes (table 1). 159/787 (20.2%) patients did not have any HH-related gene mutations. ‘Incorrect coding’ was attributed to 51/787 (6.5%) of patients. Table 3 details the diagnosis or genotype for all the patients identified from the HES APC database and whether they have undergone venesection in the past (X36.2 present or absent).

A large proportion of patients with C282Y homozygosity (86.8%) and compound heterozygosity (75.7%) have undergone at least one treatment with venesection in the past 5 years. A total of 16 patients with either C282Y heterozygosity, H63D heterozygosity or H63D homozygosity have been treated with venesection. 32.9% (24/73) of patients diagnosed with hyperferritinaemia have undergone prior venesection. Almost all (45/51) labelled as ‘incorrect coding’ did not undergo any venesection.

### Algorithms

Table 4 shows the performance of each algorithm generated with algorithms 4 and 6 providing the best PPVs at 74.3% and 74.4%, respectively, in identifying patients with HH. Algorithm 6 also provides the best specificity at 71.1% but does result in a significant drop in sensitivity compared with other algorithms. When using algorithm 4 (table 5), of the 142 non-C282Y homozygotes but positive for haemochromatosis (false positives), 8 patients had non-HH conditions which require venesection, 6 patients were incorrectly coded, 78 were compound heterozygotes, 30 had other HH genes and 20 were categorised as hyperferritinaemia. Among the 68 false negatives (table 5), 2 patients also had leukaemia on top of an HH diagnosis and 3 patients were incorrectly coded as polycythaemia.

**Table 4:** Performance of different algorithms to identify haemochromatosis patients with C282Y homozygosity

Algorithm	Sensitivity (%) and 95% CI	Specificity (%) and 95% CI	PPV (%) and 95% CI	NPV (%) and 95% CI
1	98.7 (97.3 to 99.5)	11.0 (7.8 to 15.1)	63.3 (59.7 to 66.8)	85.0 (70.2 to 94.3)
2	86.8 (83.5 to 89.7)	46.4 (40.8 to 52.2)	71.6 (67.7 to 75.2)	69.4 (62.6 to 75.6)
3	86.2 (82.8 to 89.2)	50.3 (44.6 to 56.0)	73.0 (69.1 to 76.6)	70.1 (63.6 to 76.1)
4	85.8 (82.4 to 88.8)	53.9 (48.2 to 59.6)	74.3 (70.5 to 77.9)	70.9 (64.7 to 76.7)
5	76.4 (72.3 to 80.1)	55.8 (50.1 to 61.5)	72.9 (68.8 to 76.8)	60.4 (54.4 to 66.1)
6	53.9 (49.3 to 58.4)	71.1 (65.7 to 76.1)	74.4 (69.4 to 78.9)	49.8 (45.0 to 54.5)

NPVnegative predictive valuePPVpositive predictive value

**Table 5:** 2×2 contingency table to identify C282Y homozygotes using algorithm 4

	C282Y homozygosity present	Non-C282Y homozygotes	Total
Positive for haemochromatosis	411	142	553
Negative for haemochromatosis	68	166	234
Total	479	308	787

## Discussion

This study demonstrates the utility of an algorithm to improve the identification of haemochromatosis patients with C282Y homozygosity using the HES APC database. This is the first set of algorithms to be described for identifying this group of patients from EPR using inpatient coding. The heterogeneity of diagnosis/genotype in the baseline population reflects the challenge of using the ICD-10 code E83.1 with only 60.9% (479/787) identified as having haemochromatosis based on our study’s definition. Those aged ≥60 with E83.1 were less likely to be C282Y homozygotes, which could be attributed to the increasing prevalence of conditions associated with hyperferritinaemia and multimorbidity.^22^

The application of the OPCS-4 code X36.2 (algorithm 2, 3, 4, 5 and 6) significantly improves the specificity in recognising C282Y homozygotes. Also, a PPV of >70% is seen in all five algorithms that use the procedure code X36.2. When individuals with two copies of the C282Y gene develop iron overload evidenced by raised iron indices with or without organ dysfunction, most will undergo the induction phase of venesection which requires weekly to fortnightly treatment.^6 21^ These frequent treatment episodes lead to recurrent generation of inpatient codes thus improving the reliability of identifying HH patients. A drop in sensitivity is seen with the addition of X36.2 reflecting a proportion of C282Y homozygotes who have not required venesection during the study period. This could be attributed to patients undergoing maintenance venesection with NHS Blood and Transplant as regular blood donors.^21^

Polycythaemia vera, secondary polycythaemia and porphyria cutanea tarda are treated with venesection hence attracting the X36.2 procedure code.^23 24^ 18 patients with these conditions were identified in the baseline cohort indicating coding errors due to sharing the same treatment modality as HH. An improvement in specificity and PPV is seen when patients with ICD-10 codes for these conditions are removed (algorithm 3).

A further improvement in specificity and PPV occurs when patients with ICD-10 codes in G2 are excluded (algorithm 4). Transfusion-related iron overload was seen in 17 patients in the baseline cohort highlighting the inability of the E83.1 code to discern between primary and secondary iron overload. Only six patients with secondary iron overload underwent venesection while the remainder are likely to have been treated with oral iron chelation therapy.^25^ There were only modest improvements in specificity and PPV when using algorithms 3 and 4 compared against algorithm 2 in our study. However, these algorithms are likely to show larger improvements in performance when applied to datasets in tertiary hospitals that have large haematology centres providing treatment for conditions that require frequent blood transfusions such as thalassaemia and sickle cell disease or polycythaemia.

With algorithms 5 and 6, the specificity improves further by including only patients who have undergone at least 5 or 10 episodes of venesection, respectively, during the study period. This reflects the treatment approach for HH whereby patients will require regular venesections in both the induction and maintenance phases over a 5-year period thus improving the reliability. Algorithm 6 provides the best specificity; however, there is a large drop in sensitivity compared with algorithms 1–4 highlighting patients who are donating blood as part of their maintenance phase and are not captured by the HES APC database. Some HH patients are also not identified by algorithms 5 or 6 due to non-compliance with treatment and being lost to follow-up in secondary care. Additionally, it may be that as patients become older and frailer, their venesection treatments are stopped following discussion about the risks and benefits of ongoing treatment.

Algorithm 6 has the highest PPV, but algorithm 4 may be the preferred optimised algorithm to diagnose haemochromatosis patients with C282Y homozygosity within a large data set of inpatient records as it has the second highest PPV while providing a good balance of sensitivity and specificity. In addition, research that seeks to understand the impact of HH on patients and the healthcare system, such as healthcare utilisation and assessing geographical variation in outcomes would also benefit from using these algorithms to identify patients more reliably within data sets. Future studies reviewing chronic liver disease, liver cirrhosis and hepatocellular carcinoma from national data sets will be better able to analyse their findings using any of the algorithms as they provide a better PPV compared with the current practice of relying only on the diagnosis code E83.1.^17^,^19^ To screen for C282Y homozygotes, algorithm 1 would suit best with an excellent sensitivity of 98.7% ensuring almost all these patients are captured.

The specificity for all algorithms is at best approximately 70% highlighting the number of patients without C282Y homozygosity who may be inappropriately receiving venesection. 27.4% (20/73) of patients with hyperferritinaemia are still included by the optimised algorithm 4 highlighting that these patients are undergoing venesection without a formal diagnosis of iron overload disorder. A French study of 1059 patients undergoing venesection identified that just 24% were C282Y homozygotes and 14% compound heterozygotes with a large proportion of the remainder having metabolic syndrome, obesity or excess alcohol consumption.^26^

### Strengths and limitations

Currently, there are no published studies describing methods that can accurately identify HH patients within EPR using coding. The strength of this study is the case note review of all included patients which serves as the gold standard to determine the various causes of iron overload. In addition, this study benefits from the methodical generation of algorithms taking into account the various factors that influence the E83.1 diagnosis code. As a result, the generated algorithms provide improved performance in identifying haemochromatosis patients with C282Y homozygosity compared with solely using the diagnosis code E83.1 The preferred optimised algorithm (algorithm 4) is a valid algorithm to use in England as the HES APC database will often capture venesection treatment episodes. This algorithm can also be applied to large data sets in healthcare settings that use similar systems of coding. Healthcare systems that have robust outpatient coding can use this algorithm as well.

In certain UK centres, venesection is undertaken in an ‘outpatient’ setting. As a result, these treatment episodes will not be coded in the HES APC database and patients can be missed. Including outpatient databases may not necessarily yield better results due to their unreliability.^20^ Some patients also regularly donate blood with NHS Blood and Transplant as part of the maintenance phase of their treatment; hence, these treatment episodes will not be coded in the HES APC database. In addition, patients who are treated only with oral iron chelation will not be captured either. Our study methods and results would benefit from external validation with a larger cohort of patients especially in a tertiary centre that provides care for more patients who are susceptible to transfusion-related iron overload.

## Conclusion

These novel algorithms represent a more reliable method to detect haemochromatosis patients with C282Y homozygosity from EPR than the single ICD-10 code E83.1. It may be applied to large administrative data sets for investigation of HH in population studies.

## supplementary material

10.1136/bmjopen-2024-089369 online supplemental file 1

## Data Availability

No data are available.
