# Supplementary material for: Identifying haemochromatosis patients with C282Y homozygosity from inpatient electronic patient records in England using a novel algorithm: a retrospective observational study
Source: BMJ Open. 2025 Feb 18;15(2):e089369. doi: 10.1136/bmjopen-2024-089369 (PMC11836853; doi:10.1136/bmjopen-2024-089369)
Supplement: online supplemental file 1 [file bmjopen-15-2-s001.docx]

**Supplementary Table 1: Demographic characteristics of patients with ICD-10 code E83.1 in Y&S cohort**

| **Characteristic** | **Total (n=787)**  **N(%)** | **C282Y homozygous (n=479)**  **N(%)** | **Non-C282Y homozygous (n=308)**  **N(%)** |
| --- | --- | --- | --- |
| **Age group** |  |  |  |
| <40 | 74 (9.4) | 52 (6.6) | 22 (2.8) |
| 41-50 | 85 (10.8) | 67 (8.5) | 18 (2.3) |
| 51-60 | 158 (20.1) | 106 (13.5) | 52 (6.6) |
| 61-70 | 193 (24.5) | 116 (14.7) | 77 (9.8) |
| 71-80 | 164 (20.8) | 80 (10.1) | 84 (10.7) |
| 80+ | 113 (14.4) | 58 (7.4) | 55 (7.0) |
| **Sex** |  |  |  |
| Male | 460 (58.4) | 269 (34.1) | 191 (24.3) |
| Female | 327 (41.6) | 210 (26.7) | 117 (14.9) |
