# Supplementary material for: Identifying haemochromatosis patients with C282Y homozygosity from inpatient electronic patient records in England using a novel algorithm: a retrospective observational study
Source: BMJ Open. 2025 Feb 18;15(2):e089369. doi: 10.1136/bmjopen-2024-089369 (PMC11836853; doi:10.1136/bmjopen-2024-089369)
Supplement: Supplementary file 2 [file bmjopen-2024-089369.reviewer_comments.pdf]

PEER REVIEW HISTORY

BMJ Open publishes all reviews undertaken for accepted manuscripts. Reviewers are asked to complete a checklist review form and are provided with free text boxes to elaborate on their assessment. These free text comments are reproduced below.

ARTICLE DETAILS

Title (Provisional)

Identifying haemochromatosis patients with C282Y homozygosity from inpatient electronic patient records in England using a novel algorithm: a retrospective observational study

Authors

Singh, Prabhsimran; Millson, Charles; Huang, Chao; Driver, Robert J

VERSION 1 - REVIEW

**Reviewer**	**1**
**Name**	**Savatt, Juliann**
**Affiliation**	**Geisinger, Department of Genomic Health**
**Date**	**24-Jul-2024**
**COI**	**No conflicts to disclose**

Thank you for the opportunity to review this manuscript. Singh et al, summarize creation and validation of algorithms leveraging electronic patient records to identify patients with symptomatic hereditary hemochromatosis (HH). The use of electronic patient record data is valuable to health-related research; as the authors point out, there are variable practices in medical coding and the success of identifying relevant patients using coding data can be variable. Creation of identification algorithms with sensitivity/specificity/PPV/NPV is valuable. I believe the manuscript could be strengthened with some modifications.

The authors have focused on identifying patients who are clinically diagnosed with HH. Studies have suggested that a proportion of patients with HH have yet to come to clinical attention. The research question should be clarified to note that the algorithms created are focused on those with a diagnosis. Further rationale of why such identification is important would be useful as well. For instance, does identification improve population level tracking of HH and outcomes?

Within the methods, a clearer description of how false positives and false negatives were determined would be valuable. Who completed the review of case notes and how were patients categorized? A figure describing categorization of patients in methods could be useful to the reader. I believe the authors noted those who are C282Y homozygous as true

positives and all others as false positives. Is that correct? If so, the purpose of the manuscript was to identify those with clinically symptomatic C282Y homozygosity and not symptomatic HH. As the authors point our compound heterozygous C282Y/H63D are at risk for iron overload and other genetic causes of HH. Clarification and revision are important to articulate the goal of the algorithm m, the methods, and the results, and conclusions/limitations more clearly.

A major weakness of the study is that the study did not evaluate the performance of the baseline algorithm. How many patients does that algorithm miss with HH? To truly understand the ability for these algorithms to identify patients with HH, it seems to be important to explore performance of the baseline algorithm.

Some of the algorithms relied on performance of venesection. Maintenance phlebotomy frequency varies and can be less frequent (~2 times per year). Did the authors consider other treatments such as chelating agents?

The discussion would benefit from further discussion of how these algorithms could be used.

Minor Suggestions:

Within the abstract, I would suggest a few clarifications for the reader:

- What is ICD Code E83.1? While this is defined in text, defining in the abstract would be beneficial.

- EPR is not a defined abbreviation

- Clarify were those without HH gene mutations negative and tested or untested in abstract.

I do not see a clear ethics statement.

**Reviewer**	**2**
**Name**	**Brown, Kyle E**
**Affiliation**	**University of Iowa Health Care**
**Date**	**29-Aug-2024**
**COI**	**None**

This study addresses the limitations of ICD-10 code E83.1 to accurately identify patients with HH. The authors found that combining documentation of C282Y homozygosity with exclusion of non-HH condition that are treated with venesection and exclusion of hematologic conditions associated with transfusional iron overload PLUS a procedure code for venesection yielded the best positive predictive value for HH of the algorithms they

tested. They suggest that this algorithm could be used in large datasets to investigate HH in population studies.

This work addresses a difficulty in studies of HH, in that the specificity of ICD coding for HH is very poor. The proposed algorithm could facilitate identification of "real" patients for study purposes. Although the inclusion of the venesection criterion is logical, its contribution to the performance of the algorithm is minor: the sensitivity of Algorithm 4 is very similar to Algorithm 3 (which excludes the other conditions that are either treated with venesection or that cause transfusional iron overload) and the specificity of the former is only modestly improved versus the latter. Even Algorithm 5 (which includes the same exclusions but specifies >5 episodes of venesection) has a specificity of <60%. The point is that patients with HH are heterogeneous (some undergoing frequent venesection for initial de-ironing, others having maintenance venesections a few times a year, and some who do not require ongoing venesection) and the heterogeneity is problematic for an approach such as this. Ultimately, whether the use of the algorithm is a sound approach for a study would depend on the question that is being researched.

It would be interesting to know how the authors settled on the frequency of >5 venesections for Algorithm 5. Given that the study period was 5 years and the fact that even in the maintenance phase of treatment, most HH patients will have more than 1 venesection/year, this is arguably a low frequency of venesections. Presumably setting the bar higher would enhance specificity but at the cost of further diminishing sensitivity?

In order to understand how well the proposed approach would function in population studies, it would be helpful to have some idea of the proportion of HH patients in the UK who have outpatient venesections (not coded in the database) or those who are blood donors.

Finally, a minor correction is needed in the first sentence of the Introduction, where the authors state that HH is an inherited disorder of haemostasis, rather than homeostasis, which is presumably what was meant.

VERSION 1 - AUTHOR RESPONSE

1. Two changes made to introduction section to clarify that this study is to identify patients with an established diagnosis of HH:
    a. Para 2 highlights the variable biochemical and clinical penetrance of haemochromatosis indicating the heterogeneity of this condition.
    b. Last paragraph clarifies that the aim of the study to identify patients with an established diagnosis of haemochromatosis using coding.

2. The methods section has been refined to provide greater clarity in who undertook case note review, how a diagnosis of haemochromatosis was determined and the stepwise process to confirm other diagnoses associated with E83.1

3. The baseline algorithm performance (E83.1 code only) is not in the table but is mentioned within the manuscript text. This has been highlighted now. The baseline algorithm predicted only 60.9% (479/787) to be homozygous for the C282Y mutation.

4. Further discussion written on the role and applicability of these algorithms in the discussion section as advised.

5. We have included the inability to identify patients being treated with oral iron chelation only in the limitations section of the text as well as the bullet point ‘strengths and limitations’ section at the top.

   None of the patients that have haemochromatosis in our study cohort are being treated/have required treatment with oral iron chelation therapy. There are no specific OPCS-4 or ICD-10 codes that refer specifically to treatment with oral iron chelation that would have allowed its application to the algorithms to further improve performance.

6. Further elaboration in manuscript text regarding only modest improvements in specificity and PPV with use of algorithm 3 and 4 as mentioned by Reviewer 2.

   We appreciate that there are only modest improvements in specificity and PPV between Algorithm 3 and 4 but this may likely have larger improvements in tertiary centres that have large haematology centres treating conditions that require frequent blood transfusions such thalassaemia and sickle cell disease.

   We also agree and appreciate the comment that the heterogeneity of this patient population provides challenges to our algorithm approach. We wanted to improve the care of our patients with HH by being able to reliably identify them from the population our NHS Trust serves which is what inspired our study. The current methods are imprecise estimates when evaluating patients with haemochromatosis in large datasets. Despite their challenges, these algorithms we feel will be valuable in specific research studies (as the reviewer pointed out) and also going into the future given the lack of specific haemochromatosis coding in ICD-11.

7. Following feedback from both reviewers, a further algorithm has been added (Algorithm 6). The reasoning for Algorithm 5 and 6 which uses frequency of venesections have been elaborated in the methods section. The performance of both these algorithms have been further critiqued in the discussion section.

   Patients requiring maintenance venesection will require at least between 2-6 venesections per year in keeping with current 2022 European Association for the Study of the Liver guidelines for the management of haemochromatosis. Taking the lower number of this, patients with haemochromatosis should have at least 10 venesections in the 5-year study period.

   We settled on 5 venesections considering the following factors: some patients can become frail hence venesections were stopped/reduced, a pause in venesection for patients who develop other medical conditions that take priority i.e. cancer treatment and patients that were non-adherent by only attending sparingly.

The reviewer did accurately predict the outcome of using a greater bar of venesection frequency. By applying an algorithm (algorithm 6) that uses ≥10 venesections, there is an increase in specificity to 71.1% compared to 58.1% in Algorithm 5. But there was a significant drop in sensitivity to 53.9% with a PPV of 74.4% which only matches the best PPV (Algorithm 4).

8. Further elaboration in the manuscript on why C282Y homozygotes only were chosen for the algorithm development is included.

   Thank you for your comments and feedback regarding our use of C282Y homozygosity only to characterise haemochromatosis. Within our introduction we refer to compound heterozygotes as being less likely to have organ dysfunction or morbidity related to HH. The UK BioBank database reviewed 10701 compound heterozygotes and did not find increased mortality in this group compared to those without any HFE mutations. Increasingly in many international guidelines including the 2022 European Association for the Study of the Liver and 2019 American College of Gastroenterology Haemochromatosis Guidelines; compound heterozygotes are acknowledged to have raised iron indices but a lower risk of morbidity and mortality as well as at risk of organ dysfunction in the presence of other cofactors only. It is recommended within the guidelines that patients with compound heterozygosity with very high ferritin levels in the absence of cofactors should undergo MRI Liver iron concentration assessment to objectively establish presence of iron overload followed by second line genetic testing to confirm their diagnosis of haemochromatosis.

   We have included **at the end of this document** for the reviewers to see the performance of all the algorithms when compound heterozygotes were included. There is improvement in PPV across all algorithms in a manner similar to using C282Y only.

In addition, the paragraphs below are our response to feedback and comments where changes have not been directly made in the manuscript:

1. We agree with the reviewer that improving specificity in this highly heterogenous group of patients is very challenging. We believe we have applied the best possible criteria that influences the E83.1 code to improve the specificity. The addition of venesection procedure code to the algorithms dramatically improves specificity from 11% in Algorithm 1 to greater than 46% in algorithm 2-5.

   The challenge in improving specificity remains that there are patients with hyperferritinaemia without haemochromatosis that are being venesected and algorithms relying on coding will struggle to improve the specificity in these cases until clinical practice changes in keeping with national and international guidelines. As our manuscript highlights in the discussion section, how in a French centre treating >1000 patients with venesection, only 24% were C282Y homozygotes with a large proportion of the remainder having metabolic risk factors.

2. We agree with the reviewers' comments and points regarding outpatient venesection. We do appreciate that an estimate of outpatient venesection and blood donation provides greater

clarity to understand the function of our algorithms and how reliable they are to identify HH patients.

Unfortunately, we are unable to provide a decent estimate of haemochromatosis patients who donate blood currently as this data is not available. Historically these patients were not allowed to use the blood donation service due to their condition, but things have changed in the last few years with the NHSBT service welcoming patients with HH to sign up, now with a dedicated phone line for patients to register. However, this is a new change, and we cannot accurately identify this group of patients currently.

Estimating OP venesection is equally challenging. A recent survey on the provision of venesection in the United Kingdom which was presented as a poster at the UK national liver conference 2024, highlighted the variability of venesection treatment coding (https://gut.bmj.com/content/73/Suppl_3/A78.2). However, it did highlight that venesection is predominantly recorded as a day case attendance or venesection treatment (47/59 NHS Trusts) which will attract appropriate OPCS-4 treatment codes.

Table 4: Performance of different algorithms to identify HH (including compound heterozygotes)

Algorithm	Sensitivity (%) and 95% CI	Specificity (%) and 95% CI	PPV (%) and 95% CI	NPV (%) and 95% CI
1	98.5	15.4	77.2	77.5
2	84.8	58.2	85.5	56.8
3	83.8	62.7	86.7	57.0
4	83.4	68.2	88.4	58.5
5	75.8	71.1	88.4	50.2
6	53.2	82.6	89.9	37.7

VERSION 2 - REVIEW

**Reviewer**	**1**
**Name**	**Savatt, Juliann**
**Affiliation**	**Geisinger, Department of Genomic Health**
**Date**	**02-Jan-2025**

COI

Thank you for the opportunity to rereview this manuscript. Singh et al, summarize creation and validation of algorithms leveraging electronic patient records to identify patients with symptomatic hereditary hemochromatosis (HH). The revised manuscript is much increased in clarity.

A remaining concern I have upon review is that authors fail to communicate that the algorithm intends to identify symptomatic C282Y homozygous individuals not symptomatic HH. This should be made clearer to the reader throughout the manuscript (e.g., Table titles, Title, etc) as it has implications for the use of these algorithms. For example, these algorithms could be used to assess symptomatic C282Y in large datasets but would have unknown utility in identifying all individuals with C282Y homozygosity or all individuals with symptomatic HH.

Minor Comments

• The abstract would benefit with more description of the other data points leveraged in the algorithms.

• Strengths and limitations: Expand on the limitation that the algorithm relied on inpatient venesections and, as such, did not capture outpatient performance of this therapy. From these bullets, the reader does not understand that only inpatient venesections were used.

• Strengths and limitations: Abbreviations are not defined and should be expanded.

• Introduction: The last paragraph should be incorporated into the methods and discussion rather than the introduction.

• The presence of cofactors such as alcohol excess and Mutations to other genes (non HFE) that cause iron overload such as haemojuvelin (HJV), hepcidin antimicrobial peptide (HAMP), transferrin receptor 2 (TFR2) and SLC40A1 (ferroportin disease) are all rare. 6 – "Mutations" is erroneously capitalized"

• Results – Was "best PPV" defined based on PPV and CI? If looking just at PPV, algorithm six is higher?

VERSION 2 - AUTHOR RESPONSE

I have detailed specific areas below where changes have been made based on the reviewer's minor comments:

1. Abstract: Further additions to results section of the abstract as advised
2. Strengths and Limitations: Limitation of study in relation to using only inpatient venesection episodes described in this section now as advised
3. Strengths and Limitations: Abbreviations in this section now defined

4. Introduction: Last paragraph removed and incorporated into methods section
5. Typographical area highlighted in introduction section now edited
6. Results: Changes made to manuscript in regard to interpreting the results of algorithm performances for best PPV. The discussion section was updated as well taking into account these changes.

One additional change has been made to table 3 whereby several of the rarer genes have been combined into one category called 'Rarer mutations' to prevent patient identification given the small numbers within the previous categories.

Additionally, the reviewer highlighted a remaining concern regarding the purpose of our study, as we have defined haemochromatosis for the purpose of this study to be those with C282Y homozygosity only. We appreciate the concerns raised and have taken this into account by making various changes throughout the manuscript including the title, abstract, tables and discussion to reflect that the intention and performance of algorithms is focused on identifying those with C282Y homozygosity and a diagnosis of haemochromatosis – the group with the highest risk of morbidity and mortality related to HFE mutations.

We agree that these algorithms have an unknown utility to identify all C282Y homozygotes. Given the pathophysiology of haemochromatosis, that has a variable biochemical (raised iron indices) and clinical penetrance (organ dysfunction) not all patients with 2 copies of C282Y homozygosity will develop iron overload or have iron overload during the period of a study. This group is then unlikely to be identified within large inpatient datasets as they have not required treatment due to having normal indices, which is the main trigger for clinicians to usually initiate treatment. It is when evidence of iron overload emerges either through raised iron indices or organ dysfunction, that the term 'haemochromatosis' is applied rather than solely based on genetic make-up. As such, we have used the term **"haemochromatosis patients with C282Y homozygosity"** or a variation of this to reflect the intention of our study and hopefully alleviate the concerns raised.

Although, as with studies relying on coding, there will remain limitations and a small amount of patients with C282Y homozygosity without iron overload being coded as E83.1. For example, a woman with C282Y homozygosity identified through family screening may attract the E83.1 code during an inpatient stay for giving birth although never showing any evidence of iron overload thereby existing within a dataset and influencing the sensitivity, PPV etc when algorithms are applied.
